# Altered motor learning and coordination in mouse models of autism spectrum disorder

**DOI:** 10.3389/fncel.2023.1270489

**Published:** 2023-11-08

**Authors:** Katherine R. Cording, Helen S. Bateup

**Affiliations:** ^1^Helen Wills Neuroscience Institute, University of California, Berkeley, Berkeley, CA, United States; ^2^Molecular and Cell Biology Department, University of California, Berkeley, Berkeley, CA, United States; ^3^Chan Zuckerberg Biohub, San Francisco, CA, United States

**Keywords:** striatum, motor learning, autism spectrum disorder, mouse models, corticostriatal, rotarod, direct pathway, indirect pathway

## Abstract

Autism spectrum disorder (ASD) is a complex neurodevelopmental disorder with increasing prevalence. Over 1,000 risk genes have now been implicated in ASD, suggesting diverse etiology. However, the diagnostic criteria for the disorder still comprise two major behavioral domains - deficits in social communication and interaction, and the presence of restricted and repetitive patterns of behavior (RRBs). The RRBs associated with ASD include both stereotyped repetitive movements and other motor manifestations including changes in gait, balance, coordination, and motor skill learning. In recent years, the striatum, the primary input center of the basal ganglia, has been implicated in these ASD-associated motor behaviors, due to the striatum’s role in action selection, motor learning, and habit formation. Numerous mouse models with mutations in ASD risk genes have been developed and shown to have alterations in ASD-relevant behaviors. One commonly used assay, the accelerating rotarod, allows for assessment of both basic motor coordination and motor skill learning. In this corticostriatal-dependent task, mice walk on a rotating rod that gradually increases in speed. In the extended version of this task, mice engage striatal-dependent learning mechanisms to optimize their motor routine and stay on the rod for longer periods. This review summarizes the findings of studies examining rotarod performance across a range of ASD mouse models, and the resulting implications for the involvement of striatal circuits in ASD-related motor behaviors. While performance in this task is not uniform across mouse models, there is a cohort of models that show increased rotarod performance. A growing number of studies suggest that this increased propensity to learn a fixed motor routine may reflect a common enhancement of corticostriatal drive across a subset of mice with mutations in ASD-risk genes.

## Introduction

An estimated 1 in 100 children globally have autism spectrum disorder (ASD), and CDC estimates indicate even greater prevalence in America, where roughly 1 in 36 children is diagnosed with ASD (Zeidan et al., 2022; Maenner et al., 2023; Talantseva et al., 2023). As ASD is highly heritable (Sandin et al., 2017), much work has been done in recent years to identify genes that confer risk of developing ASD. Increased accessibility of DNA sequencing has allowed for the identification of hundreds of ASD risk genes, which range widely in the types of proteins for which they code (Satterstrom et al., 2020). Despite this molecular heterogeneity, ASD is still diagnosed through identification of behaviors that fall into two primary domains: deficits in social communication and interaction, and the presence of restricted, repetitive patterns of behavior (RRBs) (APA, 2022).

In individuals with ASD, RRBs can span a range of “lower order” and “higher level” behaviors. “Lower order” motor presentations may include self-stimulation or self-injury like head banging, hand flapping, twirling, lining up or manipulating objects, or repeatedly pressing buttons. “Higher level” repetitive behaviors include rituals, perseverative interests and insistence on sameness in a variety of situations (Caldwell-Harris, 2021). In addition to the repetitive behaviors recognized as core ASD symptoms, other motor presentations can include changes to gross motor skills such as balance, gait and posture, as well as alterations in fine motor skills and motor skill learning (Chukoskie et al., 2013). In studies of balance, individuals with ASD exhibit reduced postural control, in particular when somatosensory or visual challenges are introduced. This could occur when a subject is instructed to close their eyes, stand on one leg, or balance on a swaying platform, for example (Minshew et al., 2004; Travers et al., 2013). Atypical gait, which several studies have reported in individuals with ASD, may occur as a result of difficulties with balance and posture (Chukoskie et al., 2013). While specific changes in gait parameters are heterogenous across studies, a lack of smoothness, irregular trunk movements, and shorter stride length are commonly identified in individuals with ASD (Vernazza-Martin et al., 2005; Weiss et al., 2013). Foundational motor movements such as reaching and grasping have also been shown to be altered in children with ASD (Haswell et al., 2009; David et al., 2012), which may underlie some of the deficits seen in executing gross motor skills like throwing and catching, as well as fine motor skills like buttoning, manipulating small objects, and handwriting (Green et al., 2009; Chukoskie et al., 2013; Battah et al., 2023). Notably, handwriting has been reported to be significantly altered in those with ASD since the earliest descriptions of the disorder (Asperger, 1991). Although the early presence of motor symptoms is highly predictive of later overall ASD symptom severity, this remains an understudied and undertreated symptom domain (Troyb et al., 2016; Zampella et al., 2021). The use of common behavioral assays in tractable animal models of ASD can greatly assist in the identification of circuits that may underlie motor changes in autism.

Increasingly, the basal ganglia, and in particular the striatum, has been implicated in the manifestation of repetitive behaviors in ASD, because of the role of these circuits in motor learning, action selection, and habit formation (Fuccillo, 2016). Indeed, both structural and functional imaging studies identify aberrant striatal morphology and connectivity in individuals with ASD, in some cases strongly correlating with the presentation of repetitive behaviors (Hollander et al., 2005; Estes et al., 2011; Dichter, 2012). Magnetic resonance imaging (MRI) studies in mice support these findings, where a diverse range of genetic ASD mouse models exhibit altered striatal morphology and connectivity (Portmann et al., 2014; Ellegood et al., 2015; Lai et al., 2016; Wang et al., 2016). In this review we will discuss the relationship between striatal function and motor performance in mouse models of ASD, which has been illuminated through the use of a common behavioral assay of motor coordination and learning, the accelerating rotarod.

## Mouse models

An increase in the identification of genes implicated in ASD risk paired with the genetic accessibility of animal models has allowed for the development of many genetic mouse models of ASD (Bey and Jiang, 2014). Targeting mutations in these mouse models to risk genes that have been identified in individuals with ASD provides construct validity (where the perturbation used to generate the disease model recapitulates the known etiology of the disease in people) (Nestler and Hyman, 2010). Face validity of these models (where the model displays key clinical manifestations of the disease) is more challenging to achieve given the heterogeneity and variability of ASD presentations in people. That said, a range of assays have been developed with the goal of measuring mouse behaviors analogous to those comprising the symptom domains of ASD (Bey and Jiang, 2014).

For the RRB domain of ASD, mouse behavioral assays primarily fit into the “lower order” and “higher level” domain distinctions detailed above. The former is typically measured with the open-field assay, allowing for detection of changes in general locomotor features such as speed and distance traveled, as well as the presence of motor stereotypies such as repetitive grooming, rearing, circling or jumping (Gandhi and Lee, 2020). Other assays like the marble burying test and the hole board take advantage of natural exploratory mouse behaviors like digging and head poking to detect increased repetition of these spontaneous behaviors (Bey and Jiang, 2014). More complex, “higher level” aspects of RRBs can also be assessed in mice, measuring resistance to change, cognitive inflexibility and perseveration in a range of reversal learning, set-shifting and response extinction tasks (Gandhi and Lee, 2020). The changes to gross motor function and coordination that appear to coincide with the repetitive behavior domain in individuals with ASD can also be assessed in mice using balance beams and commercially available systems for measuring and analyzing gait parameters (e.g., DigiGait, Neurocube) (Simmons et al., 2021). The recent development of deep-learning-based platforms such as DeepLabCut and MoSeq allows for unsupervised, data-driven detection and analysis of mouse behavioral parameters (Mathis et al., 2018; Wiltschko et al., 2020).

One behavioral assay commonly utilized in mouse models, the accelerating rotarod task, can be used both as a measure of gross motor coordination, as well as motor skill learning. Below we will outline the structure and parameters of the rotarod task, the way that learning occurs over the course of trials, and the brain regions and circuits implicated in rotarod performance.

## The rotarod task measures motor coordination and learning

**Figure 1:**
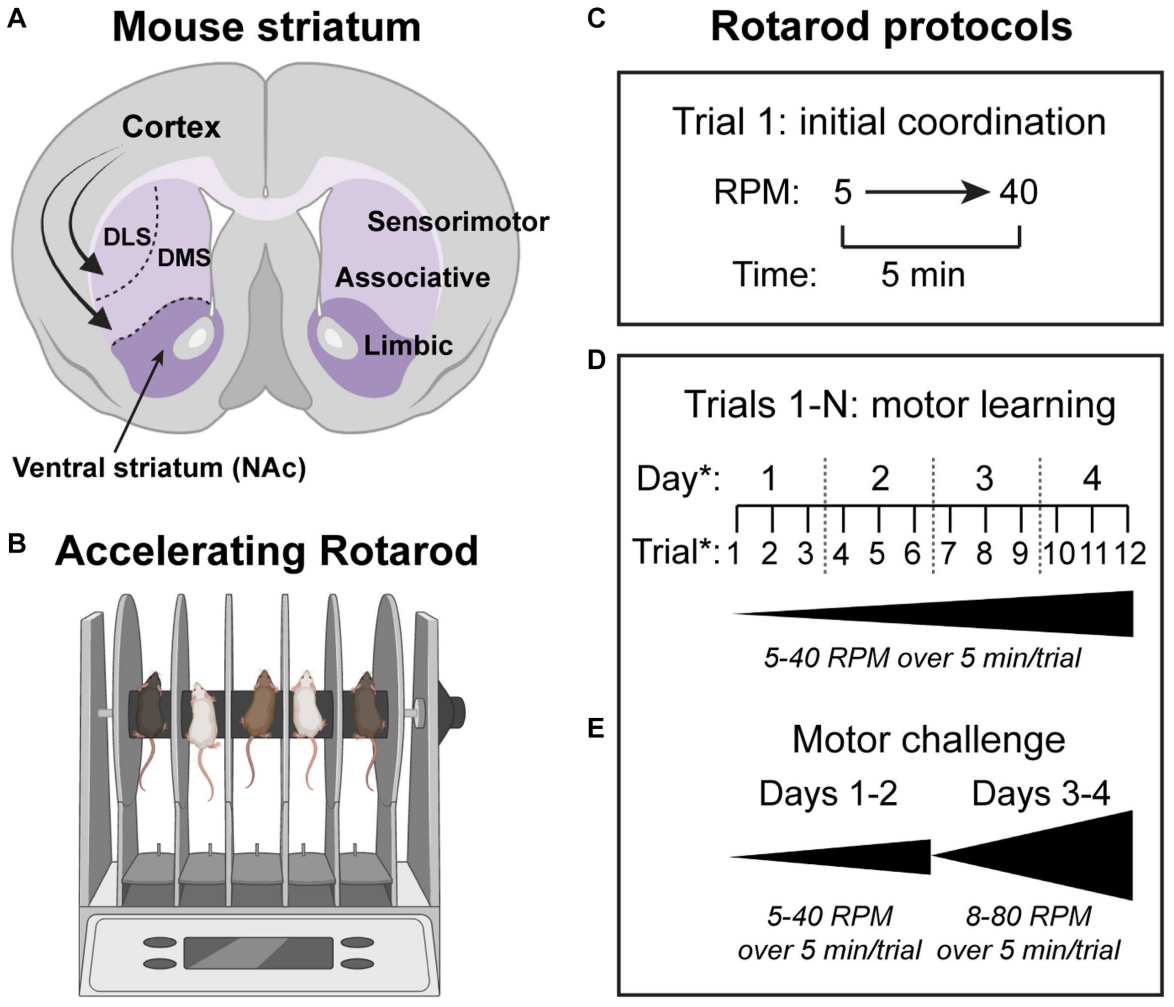
Striatal circuits drive motor learning in the accelerating rotarod task. **(A)** Schematic of a coronal mouse brain section showing the major subdivisions of the striatum (purple). DLS = dorsolateral striatum, DMS = dorsomedial striatum, NAc = nucleus accumbens. Curved arrows depict glutamatergic inputs from the cortex to all striatal subregions. **(B)** Schematic of the rotarod apparatus used to measure motor coordination and motor learning in rodents. **(C–E)** Various rotarod protocols have been used. In the simplest version of the task **(C)**, the rod accelerates from 5 to 40 revolutions per minute (RPM) over the course of 5 min. The time to fall off or rotate off the rod is a measure of motor coordination. **(D)** To measure motor learning, multiple trials are used and the gain in performance from the first to last trial is assessed for each mouse. The number of trials per day and number of testing days can vary. A common version of the task uses three trials per day across four testing days. **(E)** In some cases, a more challenging version of the task can reveal phenotypes. In this protocol, the rod is accelerated from 8 to 10 RPM up to 80 RPM over 5 min. Schematics in panels **A** and **B** were created with bioRender.com.

First described in the 1950’s (Dunham and Miya, 1957), the accelerating rotarod task has historically been used as a measure of motor coordination and function in animal models of disease (Hamm et al., 1994; Heng et al., 2008; Lubrich et al., 2022) (Figure 1). However, performance on this test can also be used as a measure of motor skill learning. In the task, mice are trained to walk on a rotating rod as it increases in speed at a constant rate. Protocols utilized in the task vary, but typically the rod increases from 5 to 40 revolutions per minute over the course of 5 min. The latency to fall, or rotate backwards off the rod, is used to determine the terminal velocity in each trial, with increases in this measure indicating better performance. Over several trials, animals exhibit improvement both within a given training day, and over the course of training sessions (Luft and Buitrago, 2005). In this way, initial performance in the task can be isolated as a measure of basic motor coordination, with differences between mouse models at this early stage indicating gross motor deficits or altered baseline motor function. If initial performance is similar, but there are differences in improvement within a given training day and/or across training days, this indicates a difference in motor learning. Many different versions of this extended protocol have been used, ranging from 3 to 5 trials for 1 day up to ten trials a day for 8 days in longer versions of the task (Yin et al., 2009). Most common is to utilize 3–4 trials per day across 3–4 days of testing (Rothwell et al., 2014; Lynch 3rd et al., 2020; Benthall et al., 2021; Le Merrer et al., 2023) (Figure 1).

When given home cage access to a running wheel, animals perform better on the rotarod overall, but the rate of both intra-and intersession improvement remains the same, indicating that increasing performance in the task goes beyond gains in locomotor fitness (Buitrago et al., 2004). Instead, animals develop and optimize a sequence of movements that allows them to stay on the rod at faster speeds, which is exemplified by shifts in gait patterns across training from stepping to running (Buitrago et al., 2004). In some cases, differences in performance between models is only revealed in versions of the task that utilize faster speeds, up to 80 revolutions per minute, which necessitates even greater motor program optimization (Rothwell et al., 2014; DiCarlo et al., 2019; Lynch 3rd et al., 2020; Benthall et al., 2021).

Given the multiphasic nature of the accelerating rotarod task, several brain regions are implicated in task performance, including the cortex (Yang et al., 2009; Fu et al., 2012; Ash et al., 2021b), basal ganglia (Costa et al., 2004; Yin et al., 2009; Durieux et al., 2012), and cerebellum (Sathyamurthy et al., 2020; Simmons et al., 2021). In this review, we highlight the role of the basal ganglia, in particular the striatum, in the motor learning that occurs during rotarod training. Given the involvement of the striatum in a number of other motor learning functions, such as instrumental learning and extinction (Yin et al., 2005, 2006; Santos et al., 2015), active avoidance, response-based procedural learning (Pittenger et al., 2006), and shifting from action-outcome to stimulus–response performance (Hawes et al., 2015), altered rotarod performance, which is easily assessed in mice, likely translates into changes in these more difficult to measure corticostriatal-dependent behaviors. In this way, performance in the accelerating rotarod task is an informative indicator of the function of a frequently altered circuit in mouse models of ASD (Li and Pozzo-Miller, 2020).

## Motor learning depends on corticostriatal circuits

The striatum, the main input center of the basal ganglia, is composed of GABAergic striatal projection neurons (SPNs) and local interneurons. SPNs, which make up over 95% of striatal neurons, send their outputs to downstream nuclei *via* two largely parallel pathways. Dopamine D1-receptor expressing SPNs of the direct pathway (dSPNs) send their primary projections to the substantia nigra pars reticulata and globus pallidus internal segment (SNr/GPi) and broadly facilitate movement when activated in bulk (Kravitz et al., 2010; Gerfen and Surmeier, 2011; Tai et al., 2012). D2-receptor expressing SPNs of the indirect pathway (iSPNs) send their primary projections to the globus pallidus external segment (GPe) and generally inhibit movement or suppress competing actions when activated as a population (Kravitz et al., 2010; Gerfen and Surmeier, 2011; Tai et al., 2012; Calabresi et al., 2014). During behavior, both populations of SPNs are activated in a coordinated way to orchestrate movement and decision-making. SPNs are innervated by a variety of inputs, most notably glutamatergic input from the cortex and thalamus, and dopamine input from the midbrain (Ding et al., 2008; Doig et al., 2010; Gerfen and Surmeier, 2011). Despite overall similar cytoarchitecture, the dorsal and ventral regions of the striatum are thought to be implicated in different functions, with the former controlling motor and cognitive functions, and the latter mediating limbic functions such as appetitive behavior and reward (Voorn et al., 2004) (Figure 1).

Further parsing of striatal regions, based primarily on differences in cortical inputs, implicates the dorsomedial striatum (DMS) as an associative region involved in the initial stages of learning action-outcome pairings and the dorsolateral striatum (DLS) as a sensorimotor region involved in the acquisition of habitual or procedural behaviors (Voorn et al., 2004). In both subregions, SPN ensemble activity and plasticity at striatal synapses is important for a variety of learning tasks, including motor skill learning (Costa et al., 2004; Barnes et al., 2005; Dang et al., 2006; Yin et al., 2009; Kupferschmidt et al., 2019). In the accelerating rotarod task, *in vivo* electrophysiological recordings showed that neurons in the striatum exhibit task-related activity that is highly correlated with performance (Costa et al., 2004; Barnes et al., 2005). Within the striatum, different subregions exhibit dynamic activity patterns throughout different phases of motor learning. In the DMS, positive modulation of firing rate in task-related SPNs predominantly occurs early in rotarod training, while in DLS, this firing rate modulation occurs after extensive training. Consistent with this, lesions of the DMS impair early learning while lesions of the DLS impair both early and late learning (Yin et al., 2009). Together this work establishes a key role for dorsal striatal circuits in rotarod learning.

While initial work highlighted the importance of the dorsal striatum in motor skill learning, several studies suggest that the ventral striatum may also play a role. In particular, a recent study showed that ablation of iSPNs in the nucleus accumbens (NAc) is sufficient to impair rotarod learning (Le Merrer et al., 2023). In addition, as discussed below, ventral striatal-specific manipulation of some ASD risk genes is sufficient to impact rotarod performance (Rothwell et al., 2014; Platt et al., 2017). This fits within the theory first introduced by Haber and colleagues that the ventral and dorsal striatum interact dynamically over the course of learning (Haber et al., 2000). Just as varying cortical inputs form a gradient across dorsolateral and ventromedial striatum, so too do the inputs to and outputs from dopaminergic substantia nigra. Ventral striatal subregions are proposed to influence behavioral gating in dorsal striatal regions through an ascending “spiral” of information through these striatonigrostriatal connections (Haber et al., 2000; Belin et al., 2007). Dynamic changes in the activity and functional roles of different SPN subtypes across this spiral likely occur during rotarod training.

In terms of the striatal cell types involved in motor learning, studies using *ex vivo* electrophysiology showed that D2-receptor expressing iSPNs of the DLS undergo significant synaptic potentiation during late training and that administration of a D2R antagonist late in training impairs rotarod performance (Yin et al., 2009). This suggests that plasticity of dorsal striatal indirect pathway activity may be important for rotarod learning. A study using adult neurotoxin-induced ablation of iSPNs throughout the striatum confirmed the importance of iSPNs for rotarod performance, particularly for early learning (Durieux et al., 2012). However, it was also shown that ablation of dSPNs throughout the striatum (Durieux et al., 2012), or selectively in the dorsal striatum (Durieux et al., 2012; Le Merrer et al., 2023), impairs rotarod performance, resulting in severe motor learning deficits. This is consistent with other studies showing that manipulations of dorsal striatal dSPNs can impact rotarod performance (Benthall et al., 2021; Ma et al., 2022). In terms of the ventral striatum, Le Merrer and colleagues showed that ablation of iSPNs (but not dSPNs) in the NAc disrupts rotarod performance (Le Merrer et al., 2023). Furthermore, reducing the excitability of dSPNs in the NAc has also been shown to impair motor learning (Rothwell et al., 2014). Together these studies provide evidence that multiple striatal circuits and subregions are required for motor learning and likely play a coordinated role in motor skill acquisition and maintenance.

The differential roles of striatal sub-regions as well as SPN subtypes during different stages of rotarod learning is likely driven by changes in cortical drive (Yin et al., 2009). Indeed, intact glutamatergic corticostriatal transmission is necessary for rotarod learning. Loss of the presynaptic scaffolding protein RIM1 from corticostriatal neurons, which disrupts excitatory transmission in the dorsal striatum, impairs rotarod learning (Kupferschmidt et al., 2019). In addition, striatal-specific deletion of glutamatergic NMDARs results in a significant deficit in learning in the task (Dang et al., 2006). Taken together, these studies show that changes in the synaptic properties of direct and indirect pathway neurons, throughout dorsal and ventral striatum, shape rotarod performance throughout different stages of the task. Our emerging understanding of the synaptic and circuit mechanisms that underlie rotarod learning make it a useful assay to apply to mouse models of disease.

## Altered rotarod performance in mice with mutations in ASD risk genes

Rotarod performance has been assessed across numerous mouse models with mutations in ASD risk genes, making it a useful assay for identifying potential convergent phenotypes. In surveying the literature, we find that many (but not all) ASD mouse models exhibit altered performance in this task, which can include altered initial performance, a global change in performance, or a difference in learning rate across trials (Table 1). One challenge with making general conclusions from this assessment is that multiple different rotarod protocols have been used. While utilizing a rod that increases in speed from 5 to 40 RPM over the course of 5 min per trial is most common, the number of trials implemented per day, and the total number of days of the task vary greatly across studies. In some cases where multiple protocols have been used, mice can show changes in one version of the rotarod task but not another (Rothwell et al., 2014; DiCarlo et al., 2019; Lynch 3rd et al., 2020; Benthall et al., 2021). Therefore, if no phenotype is reported with one rotarod protocol, it’s possible that performance would be altered if the acceleration speed, number of trials, and/or number of testing days were different.

**Table 1:** Summary of rotarod performance in mouse models with mutations in ASD risk genes.

Human gene/CNV	Mouse model	Rotarod phenotype (reference)
15q11-13	patDp/+ (6.3 Mb duplication on chromosome 7)	Similar coordination, enhanced learning (Nakatani et al., 2009)
16p11.2	Del^m^ (Mills model)	Similar coordination, enhanced learning (Lynch 3rd et al., 2020; Ouellette et al., 2020) Similar coordination, deficit in learning (Yin et al., 2021)
	Dup/+ (*Sult1a1-Spn* interval)	Similar coordination, deficit in learning (Arbogast et al., 2016)
17p11.2	*Dp(11)17/+*	Similar coordination, deficit in learning (Ricard et al., 2010)
	*Df(11)17/+*	Deficit in coordination, similar learning (Ricard et al., 2010)
*ARHGAP32* (*PX-RICS*)	*PX-RICS^−/−^*	Deficit in coordination, deficit in learning (Nakamura et al., 2016)
*ARID1B*	*Arid1b* hKO	Deficit in coordination, deficit in learning (Shibutani et al., 2017)
	*Arid1b^+/−^*	Similar coordination, deficit in learning (Jung et al., 2017)
*ARX*	*Arx^(GCG)10 + 7^*	Enhanced overall performance (Price et al., 2009)
	*Arx^dup24/0^*	Increased average latency to fall across 3 trials (Dubos et al., 2018)
*ATP1A3*	*Atp1a3^+/−^*	Enhanced overall performance (Ikeda et al., 2013)
*CACNA1G*	*Cacna1g*-Arg1723His-KI^+/−^, *Cacna1g*-Arg1723His-KI^−/−^	Deficit in coordination, deficit in learning (Hashiguchi et al., 2019)
*CADM1*	*Cadm1*-KO	Deficit in coordination, deficit in learning (Takayanagi et al., 2010)
*CDKL5*	*Cdkl5^−/y^*	Similar coordination, deficit in learning (Wang et al., 2012; Gao et al., 2020; Adhikari et al., 2022) Deficit in coordination, deficit in learning (Jhang et al., 2017)
	*Cdkl5^+/−^, Cdkl5^−/−^*	Similar coordination, deficit in learning (Fuchs et al., 2018)
*CHD8*	*Chd8^+/E31T^*	Enhanced overall performance (Hulbert et al., 2020)
	*Chd8^+/−^*	Similar coordination, enhanced learning (Platt et al., 2017)
*CNTNAP2*	*Cntnap2^−/−^*	Increased performance on a single trial (Penagarikano et al., 2011) Increased latency to fall from constant speed rotarod (Dawes et al., 2018)
*CTNNB1*	*Bfc/+*	Deficit in coordination, deficit in learning (Tucci et al., 2014)
*CYFIP1*	*Cyfip1^+/tm2a(EUCOMM)Wtsi^*	Similar coordination, deficit in learning (Bachmann et al., 2019)
	*Cyfip^+/−^*	Deficit in coordination, similar learning (Domínguez-Iturza et al., 2019)
*DDX3X*	*Ddx3x^+/−^*	Similar coordination, deficit in learning (Boitnott et al., 2021)
*DLG4*	*Dlg4^−/−^*	Deficit in coordination, deficit in learning (Feyder et al., 2010)
*DSCAM*	*Dscam^del17/del17^*	Deficit in coordination, deficit in learning (Xu et al., 2011)
*DYRK1A*	mBACtgDyrk1a (186n3)	Similar coordination, deficit in learning (Souchet et al., 2014)
*EN2*	*En2^−/−^*	Similar coordination, deficit in learning (Brielmaier et al., 2012) Deficit in coordination, deficit in learning (Cheh et al., 2006)
*FOXP2*	*Foxp2* ^*R552H*/+^	Similar coordination, deficit in learning (Groszer et al., 2008) Deficit in coordination, deficit in learning (French et al., 2012)
	*Foxp2^wt/ko^*	Similar coordination, deficit in learning (Enard et al., 2009)
*FMR1*	*Fmr1^−/−^*	Similar coordination, enhanced learning (Nolan et al., 2017) Enhanced overall performance (Roy et al., 2011) Similar coordination, deficit in learning (Bhattacharya et al., 2012; Uutela et al., 2012; Li et al., 2023)
	*Fmr1* CGG KI	Similar coordination, deficit in learning (Van Dam et al., 2005)
*GABRB3*	*Gabrb3^−/−^*	Similar coordination, deficit in learning (DeLorey et al., 1998)
	p+/m-	Similar coordination, deficit in learning (DeLorey et al., 2011)
	p−/m+	Similar coordination, deficit in learning (DeLorey et al., 2011)
*IL1RAPL1*	*Il1rapl1* ^−/Y^	Enhanced overall performance (Yasumura et al., 2014)
*KDM5C*	*Kdm5c^−/y^*	Decreased performance on a single trial (Scandaglia et al., 2017)
*KIRREL3*	*Kirrel3^−/−^*	Similar coordination, enhanced learning (Hisaoka et al., 2018)
*LRRC4*	*Lrrc4^−/−^*	Similar coordination, deficit in learning (Um et al., 2018)
*MECP2*	*Mecp2-308*	Similar coordination, deficit in learning (De Filippis et al., 2010)
	*Mecp2^tm1Tam^*	Deficit in coordination, deficit in learning (Pelka et al., 2006)
	*Mecp2^tm1.1Jae^*	Deficit in coordination, deficit in learning (Morello et al., 2018)
	*Tau-Mecp2* (overexpression)	Similar coordination, deficit in learning (Na et al., 2012)
	*Mecp2^tm1.Bird^*	Similar coordination, deficit in learning (Pratte et al., 2011) Deficit in coordination, deficit in learning (Kao et al., 2015; Vogel Ciernia et al., 2017) Decreased performance on a single trial (Santos et al., 2007)
	*Mecp2^T158A^*	Deficit in coordination, deficit in learning (Goffin et al., 2011)
	*Mecp2^R168X^*	Decreased average latency to fall across 3 trials (Schaevitz et al., 2013)
	*Mecp2^R294X^*	Deficit in coordination, deficit in learning (Collins et al., 2022)
	*Mecp2^R306C^*	Decreased average latency to fall across 3 trials (Lyst et al., 2013) Decreased average latency to fall across 2–4 trials (Ebert et al., 2013)
	*Mecp2^ΔAT-hook1^*	Decreased average latency to fall across 3 trials (Xu et al., 2018)
	*Mecp2^TG^* (overexpression)	Similar coordination, enhanced learning (Collins et al., 2004, 2022; Sztainberg et al., 2015; Ash et al., 2021a,b)
*MYT1L*	*Myt1l^+/−^*	Similar coordination, deficit in learning (Wohr et al., 2022)
*NRXN1*	*Nrxn1α* KO	Similar coordination, enhanced learning (Etherton et al., 2009)
	*Nrxn1α^+/ΔExon1^, Nrxn1α^ΔExon1/ΔExon1^*	Similar coordination, enhanced learning (Xu et al., 2023)
	*Nrxn1α^+/ΔExon9^, Nrxn1α^ΔExon9/ΔExon9^*	Similar coordination, enhanced learning (Xu et al., 2023)
*NLGN2*	*Nlgn2^−/−^*	Deficit in coordination, similar learning (Blundell et al., 2009)
	*Nlgn2^+/−^*	Similar coordination, enhanced learning (Wohr et al., 2013)
*NLGN3*	*Nlgn3^−/−^*	Similar coordination, enhanced learning (Rothwell et al., 2014)
	*Nlgn3* R451C KI	Similar coordination, enhanced learning (Chadman et al., 2008; Rothwell et al., 2014; Cao et al., 2022)
	*Nlgn3^mf^*	Similar coordination, enhanced learning (Yoshida et al., 2021)
*NF1*	*Nf1^+/−^*	Similar coordination, deficit in learning (van der Vaart et al., 2011)
	*Nf1^23a−/−^*	Similar coordination, deficit in learning (Costa et al., 2001)
*NRP2*	*Nrp2^−/−^*	Similar coordination, deficit in learning (Shiflett et al., 2015)
*NTNG1*	*Ntng2^−/−^*	Enhanced overall performance (Zhang et al., 2016)
*OTUD7A*	*Otud7a^−/−^*	Similar coordination, deficit in learning (Yin et al., 2018)
*PAX5*	*Pax5^R31Q/−^*	Deficit in coordination, deficit in learning (Kaiser et al., 2022)
*PTCHD1*	*Ptchd1^−/y^*	Decreased average latency to fall across 3 trials (Ung et al., 2018)
*PTEN*	*Pten^m3m4/m3m4^*	Deficit in coordination, deficit in learning (Tilot et al., 2014)
*RAB39B*	*Rab39b^−/−^*	Decreased average latency to fall across 3 trials (Wang et al., 2023) Similar coordination, deficit in learning (Niu et al., 2020; Zhang et al., 2020)
*RELN*	*Reln* ΔC-KI	Enhanced overall performance (Sakai et al., 2016)
	*Reln^+/rl-Orl^*	Similar coordination, deficit in learning (Sobue et al., 2018) Deficit in coordination, deficit in learning (Lalonde et al., 2004)
*SCN1A*	*Scn1a^+/−^*	Similar coordination, deficit in learning (Beretta et al., 2022)
	*Scn1a^+/R1407X^*	Enhanced coordination, similar learning (Ito et al., 2013)
	*Scn1a^+/A1783V^*	Similar coordination, enhanced learning (Miljanovic et al., 2021) Decreased average latency to fall across 3 trials (Ricobaraza et al., 2019; Fadila et al., 2020)
*SCN2A*	*Scn2a^+/−^*	Similar coordination, enhanced learning (Lena and Mantegazza, 2019) Deficit in coordination, deficit in learning (Tatsukawa et al., 2019)
	*Scn2a^+/K1422E^*	Similar coordination, enhanced learning (Echevarria-Cooper et al., 2022)
*SHANK1*	*Shank1^−/−^*	Similar coordination, deficit in learning (Hung et al., 2008; Silverman et al., 2011)
*SHANK3*	*Shank3^+/E13^*	Similar coordination, enhanced learning (Jaramillo et al., 2017)
	*Shank3^+/Δ4-22^*	Similar coordination, deficit in learning (Drapeau et al., 2018)
	*Shank3^e4-9/e4-9^*	Deficit in coordination, deficit in learning (Wang et al., 2011)
	*Shank3^E13/E13^*	Similar coordination, deficit in learning (Jaramillo et al., 2017)
	*Shank3^Δ13-16/Δ13-16^*	Similar coordination, deficit in learning (Peixoto et al., 2019)
	*Shank3^fx/fx^*	Deficit in coordination, deficit in learning (Mei et al., 2016)
	*Shank3* ^InsG3680/ InsG3680^	Deficit in coordination, deficit in learning (Speed et al., 2015; Zhou et al., 2016)
	*Shank3^Δ11/Δ11^*	Similar coordination, deficit in learning (Vicidomini et al., 2017)
	*Shank3^ΔC/ΔC^*	Deficit in coordination, deficit in learning (Kouser et al., 2013)
	*Shank3^−/−^*	Deficit in coordination, deficit in learning (Yang et al., 2012)
*SLC6A3*	DAT^T356M/T356M^	Similar coordination, enhanced learning (DiCarlo et al., 2019)
*SYNGAP1*	*Syngap1^+/−^*	Deficit in coordination, deficit in learning (Nakajima et al., 2019) Deficit in coordination, similar learning (Muhia et al., 2010)
*TOP3B*	*Top3β^−/−^*	Deficit in coordination, deficit in learning (Rahman et al., 2021)
*TSC2*	*Tsc2^+/−^*	Similar coordination, enhanced learning (Benthall et al., 2021)
	*Tsc2^ΔRG^*	Similar coordination, deficit in learning (Chevere-Torres et al., 2012)
*UBE3A*	*Ube3a^m−/p+^*	Deficit in coordination, deficit in learning (Heck et al., 2008; Leach and Crawley, 2018; Sonzogni et al., 2018; Jiang et al., 1998; Miura et al., 2002; Mulherkar and Jana, 2010; Born et al., 2017; Huang et al., 2013)
	*Ube3a^m−/p-^*	Deficit in coordination, deficit in learning (Heck et al., 2008)
	*Ube3a^Genedel^*	Deficit in coordination, deficit in learning (Syding et al., 2022)
	*Ube3a^OE^* (overexpression)	Enhanced overall performance (Punt et al., 2022)
	*Ube3a^matT503A^* (gain of function)	Similar coordination, enhanced learning (Xing et al., 2023)
*WDFY3*	*Wdfy3^+/lacZ^*	Deficit in coordination, deficit in learning (Le Duc et al., 2019)

ASD risk genes/CNVs depicted and references for a given model are representative and not exhaustive. Mouse models were chosen using the Simons Foundation Autism Research Initiative (SFARI) Gene mouse models module. Mouse models of ASD risk genes designated as Category 1 (high confidence gene) or Category 2 (strong candidate gene) by SFARI’s gene scoring system were considered. More information about SFARI gene and their gene scoring system can be found at gene.sfari.org. Mouse studies that failed to detect a phenotype are not presented, nor are models that utilized cell-type specific perturbation.

With this caveat noted, we do find a group of models, including mice with loss-of-function mutations in *Mecp2*, *Shank3* and *Ube3a*, which show consistent deficits in rotarod performance (Table 1). Some of these models exhibit poor performance from the first trial of the task, exemplified by decreased latency to fall from the rod in trial 1 compared to wild-type (WT) controls, owing to baseline deficits in motor coordination (see Table 1 - models with a deficit in coordination). In other models, trial 1 performance resembles that of WT controls, suggesting intact coordination, however, the latency to fall across trials either does not increase, or increases less than WT controls, indicating a deficit in motor learning (see Table 1 - models with a deficit in learning).

In the case of many loss-of-function *Mecp2*, *Shank3* and *Ube3a* mutations, mice exhibit deficits in both initial coordination and motor learning. The phenotypes observed in these mouse models may reflect the motor deficits that occur in individuals with mutations in these genes (Chukoskie et al., 2013; Troyb et al., 2016; Caldwell-Harris, 2021). Specifically, while motor function can be quite variable across individuals with ASD as a whole, one of the core diagnostic criteria of Rett syndrome, which is caused by loss-of-function mutations in the *MECP2* gene, is the deterioration of motor function, often resulting in complete loss of mobility in patients (Chahrour and Zoghbi, 2007). Similarly, patients with Angelman syndrome, a neurodevelopmental disorder caused by mutations in the *UBE3A* gene, generally exhibit severe motor dysfunction including orthopedic and movement difficulties, walking that is stiff or jerky, and a lack of coordination or development of complex motor skills (Rotaru et al., 2020). A comprehensive clinical assessment of 17 individuals with point mutations in the *SHANK3* gene, a gene located within the 22q13.3 chromosomal region implicated in the neurodevelopmental disorder Phelan-McDermid syndrome, identified less severe motor dysfunction than typically seen in the above syndromes; however, nearly all individuals assessed exhibited hypotonia and gait abnormalities (De Rubeis et al., 2018). The identification of motor dysfunction as a common clinical presentation caused by mutations in these genes, alongside the consistently decreased rotarod performance seen in models of these syndromes lends face validity to the rotarod assay.

Notably, while phenotypic analysis of animal models of neuropsychiatric disorders often focuses on identifying deficits, there is a cohort of ASD mouse models that show increased performance on the rotarod task (Table 1). The enhanced performance in these models can either be apparent from initial testing onward or revealed over the course of training. In the remainder of this review, we will focus specifically on these “gain-of-function” cases and discuss how enhanced motor learning may reflect changes in striatal circuit function that could facilitate the development of RRBs.

## Enhanced rotarod performance in mice with ASD risk gene mutations

### Copy number variations

Many different copy number variations (CNVs) and genomic deletions, duplications or inversions, have been found in individuals with ASD (Takumi and Tamada, 2018). The 16p11.2 variant is one of the most common CNVs associated with ASD (Weiss et al., 2008). Mice with a syntenic 16p11.2 microdeletion (16p11.2 Del^m^) have been generated and shown to exhibit increased performance on the rotarod, in particular, in a version of the task that utilizes higher speeds (8–80 RPM) (Lynch 3rd et al., 2020). Another 16p11.2 microdeletion mouse model exhibits cellular changes in the striatum including an increased number of iSPNs, increased relative volume of the ventral striatum (in particular the NAc), and excitatory synapse deficits onto SPNs in the NAc. While this mouse model has gross motor alterations such as tremors and gait changes, rotarod performance is unchanged, although the higher speeds utilized in Lynch et al. were not tested (Portmann et al., 2014). Another study found that stride and stance duration in adult 16p11.2 heterozygous mice (16p11.2 Del^m^) are significantly shorter than in controls, which are features that positively correlate with increased speed (Brunner et al., 2015). These gait changes may contribute to the increased performance on the rotarod task seen in some models of this CNV.

Another CNV implicated in ASD spans the 15q11-13 region, and is most commonly identified as a duplication (Takumi and Tamada, 2018). Mice with a paternal duplication in the 15q11-13 region exhibit increased rotarod performance compared to controls, staying on the rod for significantly longer in every trial after the first, reaching near ceiling performance (Nakatani et al., 2009). Gait assessment in another model of this CNV using a transparent treadmill identified significant changes in the motor program of these mice, which may contribute to their increased performance on the rotarod (Piochon et al., 2014).

### Cell adhesion molecules

Several of the rare genetic variants that have been identified as conferring ASD risk impact synaptic cell adhesion molecules, which are proteins involved in the formation and stabilization of synaptic contacts (Betancur et al., 2009). The best characterized synaptic cell adhesion molecules implicated in ASD are those of the neurexin and neuroligin families of proteins. *Nrxn1* (neurexin 1a) mutant mice exhibit increased performance on the accelerating rotarod, to the point of near peak performance after ten trials at 4–45 RPM over 5 min (Etherton et al., 2009). This type of enhancement has been observed in another *Nrxn1* mutant mouse model as well (Xu et al., 2023). With testing over two additional trials at five times the rate of acceleration (4–45 RPM over 1 min), *Nrxn1* knockout (KO) mice continue to perform significantly better than WT mice (Etherton et al., 2009).

Multiple *Nlgn3* (neuroligin 3) mutant mouse models also exhibit enhanced performance on the accelerating rotarod, in particular at higher speeds (8–80 RPM) (Chadman et al., 2008; Rothwell et al., 2014; Yoshida et al., 2021; Cao et al., 2022). Video analysis of one such model revealed that *Nlgn3* KO mice have reduced variability in their motor performance, streamlining step location, timing, and length significantly more than WT counterparts throughout the task. Variability in these measures negatively correlates with time to fall off the rod, indicating that they represent a valid measure of acquisition of this stereotyped behavior (Rothwell et al., 2014).

Mice lacking another ASD risk gene of the neurexin family, *Cntnap2*, which encodes a cell adhesion molecule implicated in the stabilization of potassium channels, perform significantly better than WT littermates in a single-trial version of the accelerating rotarod task (Penagarikano et al., 2011), and in a constant speed rotarod task (Dawes et al., 2018). In another study of *Cntnap2^−/−^* mice, gait analysis found that KO mice are faster than WT controls. KO mice also exhibit shorter strides, which may contribute to their increased performance (Brunner et al., 2015). A few studies identified alterations in the development or function of inhibitory interneuron populations in the striatum of *Cntnap2^−/−^* mice (Penagarikano et al., 2011; Ahmed et al., 2023), a change that may alter SPN excitability and in turn the propensity to form motor routines.

*KIRREL3* is an ASD risk gene that codes for a transmembrane protein implicated in synapse formation (Martin et al., 2015). Mice with complete loss of *Kirrel3* exhibit enhanced performance on the rotarod, particularly in later trials of the task (Hisaoka et al., 2018). Loss of the ASD risk gene *IL1RAPL1* (interleukin 1 receptor accessory protein-like 1), which also encodes a protein that mediates synapse formation, results in enhanced performance on the accelerating rotarod. *Il1rapl1^−/−^* mice are able to stay on the rod significantly longer than WT controls for all six trials of the task, demonstrating significantly increased baseline coordination, as well as motor learning (Yasumura et al., 2014).

In the space surrounding synapses, extracellular matrix proteins like reelin aid in the stabilization of cell–cell interactions. Mice with a C-terminal domain mutation in *Reln*, a gene implicated in a number of neuropsychiatric disorders such as bipolar disorder, schizophrenia, and ASD, exhibit significantly enhanced performance in the accelerating rotarod (Sakai et al., 2016). At the cellular level, another study found that a protocol used to induce synaptic long-term depression (LTD) at corticostriatal synapses in WT mice instead induces long-term potentiation (LTP) in mice with homozygous loss of *Reln*. This effect is partially explained by a loss of GABAergic tone due to decreased numbers of striatal GABAergic interneurons in *Reln* mutant mice (Marrone et al., 2006). This enhanced corticostriatal excitability could underlie the increased rotarod performance seen in some *Reln* mutant models.

### mTOR regulators

The mechanistic target of rapamycin (mTOR) serves as a central signaling hub involved in cellular metabolic processes such as protein and lipid synthesis and autophagy (Saxton and Sabatini, 2017). Several genes encoding proteins involved in the mTOR pathway are ASD risk genes, and dysregulation of mTOR signaling may occur in multiple forms of ASD (Winden et al., 2018). *TSC2*, which codes for an inhibitor of mTOR complex 1 (mTORC1) signaling, is one such ASD risk gene (Curatolo et al., 2015; Davis et al., 2015). Mice with heterozygous loss of *Tsc2* have normal initial performance but exhibit increased motor learning on the accelerating rotarod (Benthall et al., 2021). Notably, increased performance is only revealed at higher rotarod speeds (10–80 RPM), as *Tsc2^+/−^* mice perform similarly to WT littermates on 5–40 RPM trials (Benthall et al., 2021). This may reflect a ceiling effect, as WT mice can often stay on the rotarod for the entire 5-min trial with speeds up to 40 RPM.

Mice with altered function of Pten, another inhibitor of mTOR signaling, also exhibit changes in rotarod behavior. While global heterozygous loss of *Pten* does not alter rotarod performance (Clipperton-Allen and Page, 2014), Kwon et al. found that conditional loss of *Pten* results in increased performance on the accelerating rotarod compared to controls. In this model, *Pten* loss occurs in a subset of cortical and hippocampal neurons (Kwon et al., 2006). *Pten* deletion in interneurons is likely not the driver of this enhanced performance, as cell-type specific loss of *Pten* in parvalbumin (PV) and/or somatostatin (SST) interneurons led to impaired rotarod performance (Shin et al., 2021). Instead, increased local and long range excitatory input onto *Pten* KO cells in sensory cortex suggests that increased excitatory drive of corticostriatal neurons could underlie increased rotarod performance (Xiong et al., 2012).

### Transcriptional and translational regulators

Neural development requires precise coordination of molecular programs and several genes involved in transcriptional and translational control are implicated in ASD (Longo and Klann, 2021). *CHD8*, which encodes the chromatin remodeling factor chromodomain helicase DNA binding protein 8, has been identified as one of the genes with the strongest association with ASD (Weissberg and Elliott, 2021). *Chd8^+/−^* mice perform significantly better than WT counterparts on the accelerating rotarod, regardless of whether mice were trained at 4–40 RPM once a day for 5 days, or three times a day for 2 days (Platt et al., 2017). Enhanced rotarod performance was also observed in a different *Chd8* mutant model (Hulbert et al., 2020). In this study, *Chd8*^+/E31T^ mice performed significantly better than WT controls on all 4 trials of both accelerating (4–40 RPM over 5 min) and steady state (32 RPM) rotarod tasks.

As discussed above, loss of the transcriptional regulator MECP2 results in Rett syndrome, which is characterized by motor deficits in people and in mouse models. However, duplication of the *MECP2* locus causes *MECP2* duplication syndrome, which is a neurodevelopmental disorder highly comorbid with ASD (Qiu, 2018). In contrast to *Mecp2* deficient mice, mice with duplication of *Mecp2* exhibit significantly enhanced performance on the rotarod task, a phenotype that has been observed in several different *Mecp2* duplication models (Collins et al., 2004; Sztainberg et al., 2015). At the cellular level, following rotarod training, *Mecp2* duplication mice have significantly more new dendritic spines, as well as more stabilized spines on layer V pyramidal neurons in the motor cortex (M1), which project to the dorsal striatum (Ash et al., 2021b). These new stabilized spines tend to be located in clusters in *Mecp2* duplication mice, a characteristic that has been associated with increased motor skill learning (Yang et al., 2009; Fu et al., 2012). Indeed, Ash et al. found that the formation and stabilization of new spine clusters is significantly correlated with increased performance on the rotarod in both *Mecp2* duplication mice and WT controls (Ash et al., 2021b).

To interrogate the molecular mechanisms driving the enhanced rotarod learning in *Mecp2* duplication mice, Ash et al. targeted Ras–ERK signaling by intraperitoneally injecting the ERK inhibitor SL327 daily preceding rotarod training. This reversed the enhanced performance of *Mecp2* duplication mice, without altering WT performance in the task (Ash et al., 2021a). Together these findings suggest that increased synaptic stability within the corticostriatal sensorimotor loop may underlie enhanced motor learning in the context of *Mecp2* duplication. Notably, mice with a loss-of-function mutation in *Mecp2* exhibit significantly decreased spine density in pyramidal cells of both motor (Tropea et al., 2009) and somatosensory cortex, as well as altered short-term structural plasticity of spines in the latter region (Landi et al., 2011). These gene dose-dependent changes in synaptic stability within sensorimotor circuitry may contribute to the opposing impact of *Mecp2* mutations on rotarod performance.

Mutations in the *FMR1* gene result in Fragile X syndrome, a neurodevelopmental disorder with high comorbidity with ASD. *FMR1* mutations alter the expression of Fragile X Messenger Ribonucleoprotein (FMRP), an RNA binding protein involved in translational control (Jin and Warren, 2003). In one *Fmr1* KO mouse model, accelerating rotarod performance is enhanced compared to WT controls across all three sessions of the task, indicating both enhanced baseline coordination as well as increased learning over trials (Roy et al., 2011). Another *Fmr1* model exhibits similar initial coordination as WT controls, but significantly increased learning across the eight trials of the rotarod task (Nolan et al., 2017). Other studies of this model identified changes in striatal endocannabinoid-mediated long-term depression (eCB-LTD) (Maccarrone et al., 2010; Jung et al., 2012), a form of synaptic plasticity altered in other genetic mouse models with enhanced performance in the rotarod task (Martella et al., 2018; Benthall et al., 2021). In the dorsal striatum, eCB-LTD is enhanced at GABAergic synapses in the context of FMRP loss (Maccarrone et al., 2010), whereas eCB-LTD at excitatory synapses in the ventral striatum of *Fmr1* KO mice is abolished (Jung et al., 2012). Taken together, this loss of LTD at excitatory synapses and enhanced LTD at inhibitory synapses may culminate in unchecked corticostriatal drive in *Fmr1* KO mice, which could underlie the convergent motor phenotype across these models.

### Dopamine and rotarod performance

Dopamine is a potent modulator of cortical and striatal synapses (Tritsch and Sabatini, 2012) and functional dopamine signaling is important for motor performance and learning (Packard and Knowlton, 2002). Mice lacking dopaminergic neurons of the substantia nigra pars compacta, leading to 90% reductions in dorsal striatal dopamine, are unable to increase performance on the rotarod over trials, a deficit that is rescued by treatment with the dopamine precursor L-DOPA (Beeler et al., 2010). Several mouse models of ASD exhibit alterations in dopaminergic function (Kosillo and Bateup, 2021). A *de novo* mutation in *SLC6A3*, which results in a T356M amino acid substitution in the gene encoding the dopamine transporter (DAT), has been linked to ASD (Neale et al., 2012). *In vitro* characterization shows that this mutation results in efflux, rather than typical influx, of dopamine when expressed, potentially leading to greater synaptic dopamine (Hamilton et al., 2013). Mice expressing one copy of the *Slc6a3* mutation perform similarly to WT controls in early training; however, T356M^+/−^ mice exhibit significantly enhanced performance in later trials of the task. DAT expression levels are normal in these mice, but striatal dopamine (DA) reuptake is impaired and increased extracellular dopamine in the striatum results in increased striatal DA metabolism and reduced striatal DA synthesis (DiCarlo et al., 2019). Appropriate regulation of extracellular dopamine is important for rotarod performance as administration of the dopamine reuptake blocker nomifensine increases performance, while the dopamine agonist apomorphine diminishes performance (Shiotsuki et al., 2010). Nomifensine increases extracellular dopamine (Cragg and Rice, 2004), while apomorphine’s action at presynaptic D2 autoreceptors suppresses dopamine release (Schmitz et al., 2002). The opposing impacts of these drugs on rotarod performance highlight the importance of proper dopamine signaling in motor learning.

## Striatal changes drive altered rotarod performance

The majority of studies assessing accelerating rotarod performance have used mouse models with global mutations in ASD risk genes; therefore, the brain region or circuit responsible for the phenotype is difficult to ascertain. As discussed above, striatal circuits have been identified as a key node in rotarod motor learning. To directly test the contribution of striatal neurons to rotarod phenotypes, conditional KO mice have been generated in which the ASD risk gene is manipulated selectively in striatal neurons. These studies have revealed a direct link between striatal function and rotarod performance.

In the case of *Nlgn3*, conditional deletion in cells of the direct, but not the indirect, pathway of the striatum results in increased rotarod performance (Rothwell et al., 2014). A deficit in inhibition specifically onto dSPNs in these mice, which is expected to enhance excitability of the direct pathway, likely contributes to their increased rotarod performance. Indeed, in WT mice, a manipulation that reduces the activity of indirect pathway cells, which would have the net effect of facilitating direct pathway activation of downstream basal ganglia nuclei, results in increased performance in the task. In addition, rotarod performance is restored to WT levels in *Nlgn3* dSPN conditional KO mice *via* expression of the potassium channel Kir2.1, which decreases dSPN excitability (Rothwell et al., 2014). This study provides compelling evidence that altered balance between the striatal direct and indirect pathways can contribute to altered motor learning in ASD mouse models. Interestingly, conditional deletion of *Nlgn3* in the NAc, and not broadly in the dorsal striatum, is sufficient to recapitulate the enhanced rotarod performance (Rothwell et al., 2014). This finding supports a potentially underappreciated role for the ventral striatum in motor learning. A possible explanation for the cell type and anatomical specificity of *Nlgn3* deletion is that *Nlgn3* is preferentially expressed in dSPNs of the NAc and therefore expected to have a greater effect when disrupted in these cells (Rothwell et al., 2014).

As discussed above, multiple studies of *Chd8* mouse models have identified enhanced rotarod performance (Platt et al., 2017; Hulbert et al., 2020). One such study performed gene expression analysis across brain regions in *Chd8^+/−^* mice, identifying the NAc as a region with significant gene dysregulation. Following this, Platt et al. injected *Chd8*-targeting sgRNA into the NAc in a Cas9 knock-in mouse to determine the impact of *Chd8* reduction specifically in this region. Similar to the findings in the *Nlgn3* study (Rothwell et al., 2014), reduction of *Chd8* specifically in the NAc, and not the dorsal striatum, recapitulated the increased rotarod performance seen in constitutive heterozygous mice (Rothwell et al., 2014; Platt et al., 2017). Electrophysiological assessment of these mice found that SPNs of the NAc core have increased frequency and amplitude of spontaneous excitatory synaptic currents, as well as decreased amplitude of miniature inhibitory synaptic currents, suggesting overall increased excitatory drive of SPNs in the region (Platt et al., 2017). A distinction between dSPNs and iSPNs was not made in this study.

While the above studies implicate altered NAc function as a driver of enhanced rotarod performance, a recent study identified increased motor learning in a mouse model with dorsal striatum-selective *Tsc1* loss (Benthall et al., 2021). In this study, dSPN-specific deletion of the ASD-risk gene *Tsc1* resulted in increased performance on the accelerating rotarod, in particular at higher speeds (10–80 RPM). Mice with loss of *Tsc1* in iSPNs did not exhibit changes in rotarod performance, consistent with the findings in *Nlgn3* mice (Rothwell et al., 2014; Benthall et al., 2021). The D1-Cre line utilized in this study to target dSPNs is relatively restricted to dSPNs of the dorsal striatum, sparing the majority of NAc cells (Benthall et al., 2021). This, together with the results described above, indicate that altered direct pathway activity in either the dorsal or ventral striatum is sufficient to alter motor learning.

In *Tsc1* dSPN KO mice, electrophysiology experiments revealed that *Tsc1*-KO dSPNs have increased glutamate release probability at cortical inputs, resulting in enhanced corticostriatal drive. This study also found a deficit in eCB-LTD onto *Tsc1* KO dSPNs, which may explain the change in presynaptic release probability (Benthall et al., 2021). This prominent form of striatal synaptic depression works through the release of postsynaptic endocannabinoids that act on cortical presynaptic CB1 receptors, ultimately reducing the probability of neurotransmitter release (Kreitzer and Malenka, 2008; Lovinger, 2010). Loss of eCB-LTD onto *Tsc1* KO dSPNs likely renders these cells unable to depress excitatory inputs, leading to increased corticostriatal drive over time. Interestingly, a similar deficit in eCB-LTD was identified in the dorsal striatum of a *Nlgn3* mutant mouse model that exhibits enhanced performance in the rotarod task (Martella et al., 2018).

Here we have highlighted several examples of mouse models that exhibit enhanced rotarod performance. In a few of these models, striatal-specific manipulation of an ASD-risk gene was sufficient to induce changes in rotarod motor learning. In several studies, synaptic changes were reported that are expected to enhance striatal activation, particularly increase corticostriatal drive and/or excitability of the direct pathway (Rothwell et al., 2014; Platt et al., 2017; Benthall et al., 2021). Given the importance of striatal circuits for not only motor skill leaning but also habit learning etc., it seems plausible that gain-of-function at the neural circuit level facilitates the formation of fixed motor routines or perseverative behaviors. For ASD models where motor deficits predominate, the neural circuitry underlying the presence of repetitive behaviors remains to be established. In these cases, basic motor circuits may be disrupted such that rotarod deficits arise, but gain-of-function in other motor control circuitry likely drives the emergence of RRBs, for example in the case of *Shank3* models (Drapeau et al., 2018). Further investigation into the motor phenotypes of ASD mouse models and the synaptic and circuit basis of repetitive behaviors will provide additional insight into this core aspect of ASD.

## Other considerations

### Genetic background influences rotarod performance

There are several factors beyond a targeted genetic manipulation that contribute to differences in rotarod performance, which should be considered when comparing across ASD mouse models. A study assessing 16 mouse strains from the “Collaborative Cross” (CC), a large panel of inbred mice that captures 90% of the known variation among laboratory mice, found that rotarod performance varies widely across the strains (Mao et al., 2015). Forty-five gene loci associated with rotarod performance were identified using genetic linkage analysis, many of which overlap with human GWAS-nominated genes associated with neuropsychiatric disorders including ASD and ADHD. A similar study assessing a range of behaviors across 10 inbred mouse strains also found wide variability in rotarod performance across strains (Moy et al., 2007). Two of these inbred mouse strains, BTBR T^+^tf/J (BTBR) and BALB/cByJ (BALB), have been utilized for over a decade as models of ASD, owing to their strong and consistent displays of autism-relevant behaviors, including social behavior deficits and/or repetitive behaviors or stereotypies (Ellegood and Crawley, 2015). While BTBR mice exhibit deficits in the rotarod task, BALB mice perform similarly to C57BL/6J controls (Moy et al., 2007). Since these models are inbred strains that lack known genetic abnormalities and do not recapitulate known genetic causes of human ASD, it can be difficult to link neurodevelopmental changes to ASD-like behavior. However, continued study of these inbred models that demonstrate good face validity for ASD-like manifestations may uncover their potential construct and predictive validity.

Along with genetic background, body weight also significantly impacts rotarod performance, with weight being strongly negatively correlated with performance (Mao et al., 2015). While Mao et al. found that rotarod performance between sexes within a given strain is highly correlated, females tend to have a lower body weight than males, which may alter performance (Mao et al., 2015). Given that significant sex differences exist in both the prevalence and presentation of ASD in humans (APA, 2022), it’s possible that motor coordination and learning could differ across sexes within a given ASD mouse model. Variability in these factors across studies may contribute to some of the heterogeneity in rotarod outcomes reported in the literature (see Table 1).

### Environmental risk factors

Here we have focused on rotarod performance in genetic mouse models of ASD; however, there is a growing body of work implicating exposure to certain environmental factors in the manifestation of autism, including factors that illicit an immune response (Meltzer and Van de Water, 2017). In mice, this is often modeled with a maternal immune activation paradigm. Briefly, pregnant dams are directly infected with a pathogen (e.g., influenza virus, *Escherichia coli*), or injected with a substance that mimics a pathogen to evoke a large immune response. Subsequently, significant immunological, behavioral and neurodevelopmental changes can then be observed in offspring (Careaga et al., 2017). As brain development continues after birth, models of postnatal infections and postnatal immune activation have also been shown to lead to some of these changes (Depino, 2013). While some immune activation mouse models exhibit enhanced performance on the accelerating rotarod (Carlezon Jr et al., 2019), other models have no change in performance (Wei et al., 2012), or deficits in the task (Naviaux et al., 2014).

*In utero* exposure to certain medications has also been linked to the development of ASD, including the antiepileptic and bipolar medication valproic acid (VPA) (Roullet et al., 2013). The mechanism of VPA that may increase the risk of ASD is unknown, and prenatal exposure to the drug, like other implicated environmental factors, could modify existing genetic risk (Wang et al., 2017). Mouse models of VPA exposure are typically achieved through injection of VPA into a pregnant dam roughly midway through gestation, resulting in offspring that exhibit both neurodevelopmental and behavioral alterations (Roullet et al., 2013). Rotarod phenotypes across studies of VPA models vary, with some identifying enhanced rotarod performance (Hernandez et al., 2023), and others identifying no difference from WT (Gandal et al., 2010), or a deficit in the task (Wang et al., 2018), owing potentially to variability in VPA exposure protocols.

### Genetic rat models of ASD

Finally, while this review focuses on mouse models, there is a growing body of literature on genetic rat models of ASD (Berg et al., 2021; Harris et al., 2021; Dey and Chattarji, 2022), including models with mutations in ASD risk genes linked to enhanced rotarod performance discussed above, such as *Fmr1* (Till et al., 2015; D’Elia et al., 2022; Schiavi et al., 2023) and *Nlgn3* (Thomas et al., 2017; Anstey et al., 2022). However, as genetic accessibility of rat models is a recent development, assessment of rotarod performance in these models remains to be performed. Given the more expansive behavioral repertoire of rats, and the technical benefits that their larger size affords, increased study of genetic rat models of ASD is likely to benefit the understanding of rotarod behavior and motor skill learning in the context of ASD risk gene mutations in the coming years (Ellenbroek and Youn, 2016).

### Interactions between the basal ganglia and other motor control circuits

This review highlights the ways that striatal, and in particular corticostriatal, circuits play a role in accelerating rotarod performance. However, cerebellar circuits are also implicated in motor skill learning. Different cerebellar subcircuits are implicated in early versus late stages of motor skill learning, and a number of studies suggest that cerebellar relays exist within or parallel to the cortico-basal ganglia-thalamic loop controlling motor learning (Dayan and Cohen, 2011). In the rotarod specifically, a subpopulation of excitatory cerebellospinal neurons in deep cerebellar nuclei that project to the spinal cord were identified as being necessary for learning, but not the execution, of rotarod behavior (Sathyamurthy et al., 2020). The specific inputs to these neurons are yet unclear, but they may receive direct input from the cortex and/or thalamus. In the context of ASD, the cerebellum is frequently implicated as a region of potential convergent change. Postmortem studies in humans reveal cellular and structural cerebellar abnormalities in individuals with ASD (Schumann and Nordahl, 2011; Bolduc et al., 2012; Ecker et al., 2012). In addition, mouse models that target mutation of ASD risk genes specifically to cerebellar cell types can recapitulate ASD-like phenotypes (Tsai et al., 2012; Hampson and Blatt, 2015). However, we note that in the case of cerebellar-specific ASD risk gene mutations, mouse models most often exhibit deficits in accelerating rotarod performance (Tsai et al., 2012; Reith et al., 2013; Kawamura et al., 2021; Liu et al., 2022), even in cases where constitutive mutation of the gene leads to enhanced performance (Reith et al., 2013; Platt et al., 2017). Thus, while cerebellar circuits may participate in rotarod performance, it seems unlikely that they would contribute to the enhanced rotarod phenotype seen in the mouse models described above. Rather, we posit that synaptic gain-of-function in corticostriatal circuits is more likely to drive increased motor learning in the context of ASD.

## Conclusion

As the number and heterogeneity of identified ASD risk genes continues to expand, the utilization of common behavioral assays to identify convergent phenotypes in mouse models of ASD is of great benefit. When it comes to assessing motor symptom domains of ASD in mouse models, the accelerating rotarod task has proved very useful for identifying phenotypes. The task is relatively fast and straightforward to carry out, reveals information about gross motor coordination and motor learning, and has established underlying neural circuitry. Corticostriatal circuits are key regulators of rotarod performance and are increasingly implicated as a point of convergent alteration across a range of mouse models of ASD (Li and Pozzo-Miller, 2020). If these circuits are found to consistently contribute to altered behavior, they represent a potential site for targeted therapeutics, which may be applicable across ASDs of different genetic origin.

## Author contributions

KC: Conceptualization, Funding acquisition, Writing – original draft, Writing – review & editing. HB: Conceptualization, Funding acquisition, Supervision, Writing – review & editing.
